# Targeting Endolysosomal Two-Pore Channels to Treat Cardiovascular Disorders in the Novel COronaVIrus Disease 2019

**DOI:** 10.3389/fphys.2021.629119

**Published:** 2021-01-26

**Authors:** Francesco Moccia, Sharon Negri, Pawan Faris, Angelica Perna, Antonio De Luca, Teresa Soda, Roberto Berra-Romani, Germano Guerra

**Affiliations:** ^1^Laboratory of General Physiology, Department of Biology and Biotechnology “L. Spallanzani”, University of Pavia, Pavia, Italy; ^2^Department of Medicine and Health Sciences “V. Tiberio”, University of Molise, Campobasso, Italy; ^3^Section of Human Anatomy, Department of Mental and Physical Health and Preventive Medicine, University of Campania “Luigi Vanvitelli”, Naples, Italy; ^4^Department of Brain and Behavioral Sciences, University of Pavia, Pavia, Italy; ^5^School of Medicine, Department of Biomedicine, Benemérita Universidad Autónoma de Puebla, Puebla, Mexico

**Keywords:** SARS-CoV-2, COVID-19, cardiovascular system, two-pore channels, PI(3, 5)P_2_, endolysosomal Ca^2+^ signaling, NAADP

## Abstract

Emerging evidence hints in favor of a life-threatening link between severe acute respiratory syndrome coronavirus type 2 (SARS-CoV-2) and the cardiovascular system. SARS-CoV-2 may result in dramatic cardiovascular complications, whereas the severity of COronaVIrus Disease 2019 (COVID-19) and the incidence of fatalities tend to increase in patients with pre-existing cardiovascular complications. SARS-CoV-2 is internalized into the host cells by endocytosis and may then escape the endolysosomal system via endosomes. Two-pore channels drive endolysosomal trafficking through the release of endolysosomal Ca^2+^. Recent evidence suggested that the pharmacological inhibition of TPCs prevents Ebola virus and Middle East Respiratory Syndrome COronaVirus (MERS-CoV) entry into host cells. In this perspective, we briefly summarize the biophysical and pharmacological features of TPCs, illustrate their emerging role in the cardiovascular system, and finally present them as a reliable target to treat cardiovascular complications in COVID-19 patients.

## Introduction

The dramatic outbreak of the COronaVIrus Disease 2019 (COVID-19), which is caused by the novel severe acute respiratory syndrome coronavirus type 2 (SARS-CoV-2), is imposing an unmet medical need to the scientific community. SARS-CoV-2 causes viral pneumonia and acute respiratory distress syndrome (ARDS), which may be associated with a multiorgan dysfunction resulting from the cytokine storm produced by viral infection (Huang et al., 2020; Wang et al., 2020; Zhou et al., 2020). Furthermore, SARS-CoV-2 may induce major cardiovascular complications, including myocarditis and myocardial injury, acute myocardial infarction (AMI), arrhythmia, acute coronary syndrome, venous thromboembolism and stroke (Moccia et al., 2020a; Nishiga et al., 2020; Zheng et al., 2020). Moreover, a high prevalence of pre-existing CV comorbidities, such as hypertension and coronary heart disease, has been observed among COVID-19 patients and associated with worse clinical outcome and increased risk of death (Moccia et al., 2020a; Nishiga et al., 2020; Zheng et al., 2020). Coronaviruses, including those responsible for Middle East Respiratory Syndrome (MERS) and SARS-CoV, as well as Ebola virus, exploit the endolysosomal (EL) compartment to infect host cells (Burkard et al., 2014; Falzarano and Feldmann, 2015; Chao et al., 2020). Likewise, SARS-CoV-2 uses the viral spike (S) protein to bind the membrane-bound angiotensin-converting enzyme 2 (ACE2) and then undergoes rapid endocytosis to gain access into the host cell (Coutard et al., 2020; Hoffmann et al., 2020; Walls et al., 2020). ACE2 is a metallopeptidase that plays a crucial role in the cardiovascular system by cleaving angiotensin I to produce angiotensin 1–9; this, in turn, stimulates the angiotensin II type 2 receptor (AT_2_R) to induce nitric oxide (NO) release, to reduce the vascular tone, and to protect myocardium against ischemia-reperfusion (IR) injury (Mendoza-Torres et al., 2018; Paz Ocaranza et al., 2020). ACE2 is widely expressed in cardiac myocytes, vascular smooth muscle cells (VSMCs), and vascular endothelial cells (Patel et al., 2014; Chen et al., 2020; Evans et al., 2020; Nicin et al., 2020). In agreement with these observations, SARS-CoV-2 infected endothelial cells in several vascular beds (Varga et al., 2020), including cerebral capillaries (Paniz-Mondolfi et al., 2020), and induced endothelial dysfunction associated with apoptosis (Varga et al., 2020; Yin et al., 2020). Conversely, the presence of SARS-CoV-2 in cardiomyocytes is yet to be shown, although COVID-19 may induce myocarditis and AMI. In the present perspective, we discuss the possibility of targeting two-pore channels (TPCs), which are crucial to endocytosis and EL trafficking (Chao et al., 2020; Vassileva et al., 2020), to interfere with SARS-CoV-2 entry into cardiovascular cells.

## Basic Insights Into EL Ca^2+^ Signaling: The Role of TPCs in the Cardiovascular System

The EL Ca^2+^ store comprises a plethora of organelles enriched with H^+^ and Ca^2+^, which are widely distributed across the phylogenetic tree, and includes: lysosomes, lysosome-related organelles, secretory vesicles, vacuoles, acidocalcisomes, and the Golgi apparatus (Patel and Docampo, 2010; Faris et al., 2018). The free Ca^2+^ concentration in the EL compartment may vary from 2 to 3 μM in early endosomes (EEs) up to 500–600 μM in lysosomes, and therefore emerges as the second largest Ca^2+^ store in mammalian cells after the endoplasmic reticulum (ER) (Morgan et al., 2011). Lysosomal Ca^2+^ refilling is supported by the negative (−20 mV/−40 mV, lumen positive) resting lysosomal membrane potential (ΔΨ) and is driven by the H^+^-gradient, also known as proton-motive force, established across the lysosomal membrane by the vacuolar type H^+^-ATPase (v-ATPase) (Morgan et al., 2011; Xu and Ren, 2015; Faris et al., 2018). EL Ca^2+^ uptake may be prevented by blocking the v-ATPase activity with concanamycin or bafilomycin A1 and by increasing intravesicular pH with membrane-permeant bases (i.e., NH_4_Cl) or protonophores (i.e., monensin and nigericin) (Morgan et al., 2011; Faris et al., 2018, 2019). A lysosomal Ca^2+^/H^+^ exchanger (CAX) has been detected in non-placental mammalian cells, but not in higher vertebrates (Melchionda et al., 2016). The molecular mechanism which senses the intraluminal acidic pH to drive EL Ca^2+^ refilling remains, therefore, to be fully elucidated.

**FIGURE 1:**
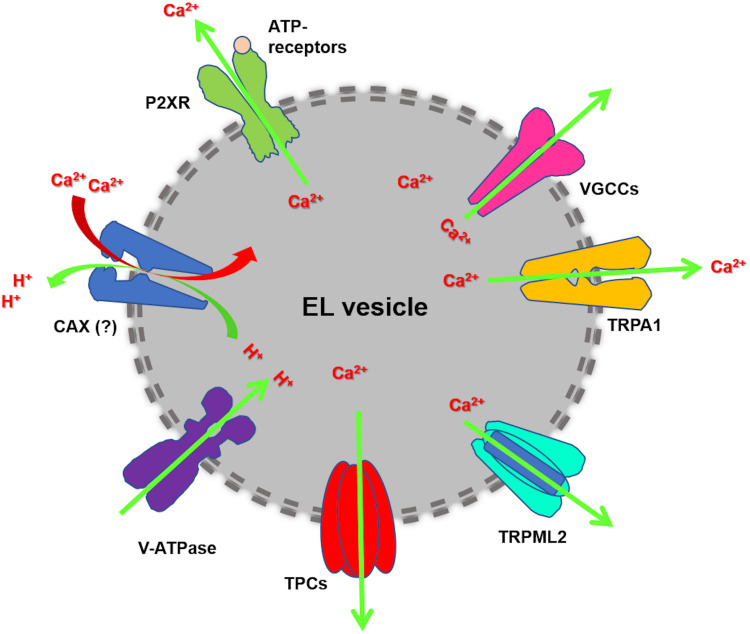
EL Ca^2+^ handling machinery. Cytosolic Ca^2+^ is sequestrated into EL vesicles by a pH-sensitive mechanism, which is maintained by the acidic intraluminal pH established through the v-ATPase. The existence of Ca^2+^/H^+^ exchanger (CAX) has been postulated in placental mammalian cells. EL Ca^2+^ mobilization may mainly occur through TPC1-2 and TRPML1. Additional EL Ca^2+^-permeable pathways can be provided by TRP Melastatin 2 (TRPM2), TRP Ankyrin 1 (TRPA1), voltage-gated Ca^2+^ channels (VGCCs) and ATP-gated ionotropic P2X receptors.

EL Ca^2+^ release may occur through multiple Ca^2+^-permeable pathways (Figure 1), including TPCs and Transient Receptor Potential Mucolipin 1 (TRPML1), TRPML2 and TRPML3 (Patel, 2015; Faris et al., 2018). TPCs belong to the super-family of voltage-gated ion channels and represent a molecular intermediate in the transition from a primordial two-domain channel, which underwent two rounds of intragenic duplication to generate, respectively, the tetrameric voltage-gated K^+^ channels (K_*V*_) and the monomeric voltage-gated Na^+^ (Na_*V*_) and Ca^2+^ (Ca_*V*_) channels (Rahman et al., 2014). The TPC gene family encompasses three members, *TPCN1*, *TPCN2*, and *TPCN3*, which, respectively, encode for TPC1, TPC2, and TPC3. However, only TPC1 and TPC2 are expressed in primates and rodents (Brailoiu et al., 2010a; Cai and Patel, 2010; Patel, 2015). TPC1 is mainly found in EEs and recycling endosomes, while TPC2 is more abundant in late endosomes (LEs) and lysosomes (Patel, 2015; Grimm et al., 2017a). Nicotinic acid adenine dinucleotide phosphate (NAADP), the most powerful Ca^2+^-releasing second messenger, has been shown to activate TPCs and mobilize EL Ca^2+^ in response to extracellular stimulation (Galione, 2015, 2019). In addition, TPCs are activated by phosphatidylinositol 3,5-bisphosphate [PI(3,5)P_2_], which is the most abundant phosphatidylinositol in LEs and lysosomes, to mediate Na^+^ and Ca^2+^-permeable channels (Jha et al., 2014). A recent investigation disclosed that TPC ion-selectivity is finely regulated by the gating stimulus: NAADP and the synthetic compound TPC2-A1-N elicit non-selective Ca^2+^-permeable currents, which lead to large increase in [Ca^2+^]_*i*_, while PI(3,5)P_2_ and the synthetic compound TPC2-A1-P activate Na^+^-selective currents, which result in weaker intracellular Ca^2+^ signals (Gerndt et al., 2020). The synthetic NAADP analog, NED-19, is widely employed to prevent NAADP binding to TPCs, thereby inhibiting NAADP-induced EL Ca^2+^ release (Patel, 2015; Pitt et al., 2016). Furthermore, molecular docking studies showed that a number of Ca_*V*_ and Na_*V*_ antagonists, including dihydropyridines (e.g., nifedipine and nitrendipine), phenylalkylamines (e.g., verapamil), and local anaesthetics (e.g., lidocaine), directly block the TPC pore and inhibit NAADP-induced Ca^2+^ release (Genazzani et al., 1997; Rahman et al., 2014). Finally, TPCs are sensitive to tetrandrine (Sakurai et al., 2015), a plant-derived bis-benzylisoquinoline alkaloid, which is widely employed in traditional Chinese medicine and may also serve as Ca_*V*_ antagonist (Yao and Jiang, 2002).

TPCs may trigger global Ca^2+^ signals evoked by a growing number of extracellular stimuli (Galione, 2015, 2019). NAADP-induced EL Ca^2+^ release may be amplified by Ca^2+^-induced Ca^2+^ release (CICR) via inositol-1,4,5-trisphosphate (InsP_3_) receptors (InsP_3_Rs) and ryanodine receptors (RyRs), possibly at junctions between acidic organelles and the ER (Galione, 2015; Penny et al., 2015; Kilpatrick et al., 2017). Ultrastructural analysis revealed that lysosomes may form close associations with the Sarcoplasmic Reticulum (SR) in both cardiac myocytes (Aston et al., 2017) and VSMCs (Kinnear et al., 2004, 2008; Fameli et al., 2014). It has been shown that β-adrenoreceptor stimulation engages NAADP-induced Ca^2+^ release through TPC2 to increase the SR Ca^2+^ load, thereby increasing SR Ca^2+^ release via RyR2 and increase cardiac contraction (Macgregor et al., 2007; Collins et al., 2011; Lewis et al., 2012). Prolonged β-adrenoreceptor stimulation may, thus, result in ventricular arrhythmia and cardiac hypertrophy in TPC2 wild-type, but not knockout, mice (Capel et al., 2015). On the other hand, TPC1 may contribute to IR injury in cardiac myocytes, by triggering the cytosolic Ca^2+^ overload and apoptotic cell death (Davidson et al., 2015). Likewise, multiple agonists, such as endothelin 1 and angiotensin II, recruit NAADP-induced EL Ca^2+^ release through TPC2 to trigger CICR through RyR3 and to promote vasoconstriction (Kinnear et al., 2004; Jiang et al., 2013; Lee et al., 2015; Trufanov et al., 2019). Ultrastructural investigations showed that TPC2 is closely apposed to RyR3 at lysosomal-ER nanojunctions, which tend to cluster in the perinuclear area and provide an ideal signaling platform to amplify the local Ca^2+^ response to extracellular stimuli (Kinnear et al., 2004, 2008; Fameli et al., 2014). Exaggerated NAADP signaling could be induced by hypoxia in pulmonary artery VSMCs and trigger the complex process of vascular remodeling that leads to pulmonary arterial hypertension (Jiang et al., 2018). Finally, TPCs are emerging as crucial players in endothelial Ca^2+^ dynamics (Moccia et al., 2019; Zuccolo et al., 2019a). NAADP activates endothelial TPCs to induce the global Ca^2+^ signals which control NO release and blood pressure (Brailoiu et al., 2010c), secretion of von Willebrand factor (vWF) and platelet aggregation (Esposito et al., 2011), neurovascular coupling (Negri et al., 2019; Zuccolo et al., 2019b; Berra-Romani et al., 2020), angiogenesis (Favia et al., 2014) and vasculogenesis (Zuccolo et al., 2016; Di Nezza et al., 2017). A recent investigation confirmed that, also in the endothelial lineage, NAADP-induced Ca^2+^ release through TPCs may be amplified into regenerative intracellular Ca^2+^ oscillations by the Ca^2+^-dependent recruitment of InsP_3_Rs (Moccia et al., 2020b).

## The Role of TPCs in the Regulation of Lysosomal Functions and Endocytosis

When TPCs are not coupled to juxtaposed RyRs or InsP_3_Rs, EL Ca^2+^ signals remain spatially restricted around the EL membrane, thereby regulating lysosomal morphology, transport, and fusion events (Grimm et al., 2017b; Lloyd-Evans and Waller-Evans, 2019; Vassileva et al., 2020). Local EL Ca^2+^ signals were shown to regulate endocytosis and vesicular trafficking of membrane receptors and protein toxins. For instance, knockout of TPC2 induced epidermal growth factor (EGF) receptor and low-density lipoprotein (LDL) receptor accumulation in LEs (Grimm et al., 2014), and delayed platelet derived growth factor (PDGF) receptor internalization and degradation (Ruas et al., 2014). Similarly, genetic deletion of either TPC1 or TPC2 induced integrin accumulation within EEs (Nguyen et al., 2017). Furthermore, knockout of TPC1 halted the uptake and trafficking through the early endocytic route of the so-called “short trip” bacterial toxins, such as diphteria and anthrax toxins (Castonguay et al., 2017). Three independent interactome screens revealed that TPCs are closely associated with Q- and R-SNARE proteins, which orchestrate intravesicular membrane dynamics (Grimm et al., 2014; Lin-Moshier et al., 2014; Castonguay et al., 2017; Krogsaeter et al., 2019). The Ca^2+^-sensor that couples EL Ca^2+^ release to endosomal membrane fusion events is yet to be identified, but could include a member of the Annexin family of Ca^2+^-binding membrane proteins (ANXA1–7), which are also part of the TPC interactome (Lin-Moshier et al., 2014; Castonguay et al., 2017). Notably, TPC-mediated local Ca^2+^ signals may also control infection and intracellular trafficking of life-threatening viral pathogens, including EBOV (Sakurai et al., 2015) and MERS-CoV (Gunaratne et al., 2018b).

## TPCs Mediate Entry and Trafficking of Viruses in Host Cells

The EL compartment provides a route that can be highjacked by viruses to penetrate into the cytosol of host cells for replication (Burkard et al., 2014; Falzarano and Feldmann, 2015; Chao et al., 2020). EBOV has long been regarded as the main causative pathogen of Ebola hemorrhagic fever or EBOV disease (EVD), which is associated with high-fatality rates in humans and primates (Feldmann and Geisbert, 2011). Following attachment to the surface of host cells, EBOV is internalized via micropinocytosis and then trafficked through EEs and LEs, where the surface viral glycoproteins (GPs) are cleaved by the lysosomal cysteine proteases, cathepsin B and L, and primed for fusion. Subsequently, viral GPs interact with their late endosomal/lysosomal receptor, protein Neimann-Pick C1 (NPC-1), which causes the viral envelope to fuse with the endosomal membrane and release the nucleocapside into the cytosol. Herein, the viral genome is transcribed and replicated, followed by the assembly of viral proteins and virions budding from the cell (White and Schornberg, 2012; Salata et al., 2019). A landmark study by Sakurai et al. (2015) revealed that genetic silencing or knockdown of either TPC1 or TPC2 inhibited EBOV infection *in vitro*. The same result was obtained by overexpressing a dominant-negative TPC2 mutant in host cells (Brailoiu et al., 2010b). The molecular deletion of TPCs interfered with virus-endosome membrane fusion and prevented nucleocapside release into the cytosol, thereby impeding viral replication (Sakurai et al., 2015). EBOV infection *in vitro* was also strongly repressed by blocking TPCs with NED-19 and with three structurally distinct inhibitors of L-type voltage-gated Ca^2+^ channels, i.e., verapamil, diltiazem, and nimodipine (Sakurai et al., 2015). These findings suggested that Ca_*V*_ antagonists, which have been approved by the Food and Drug Administration (FDA) for the treatment of multiple cardiovascular disorders (Godfraind, 2017; Oparil et al., 2018), could represent an alternative strategy to combat Ebola hemorrhagic fever. However, the Chinese medicinal herb, tetrandrine, was the most powerful inhibitor of EBOV infection *in vitro* and was therapeutically effective also in *in vivo* mouse models (Sakurai et al., 2015). Notably, tetrandrine inhibited NAADP- and PI(3,5)P_2_-evoked lysosomal currents and attenuated NAADP-induced intracellular Ca^2+^ release in living cells (Sakurai et al., 2015). A recent screening campaign confirmed that TPC2 activation regulates EBOV entry in host cells. Measurement of PI(3,5)P_2_-evoked lysosomal currents, NAADP-induced Ca^2+^ release and single-channel activity, revealed that FDA-approved dopamine antagonists, such as pimozide and fluphenazine, and selective estrogen receptor modulators, such as clomiphene and raloxifene, were also able to target TPC2 by plugging the channel pore (Penny et al., 2019). Furthermore, these novel inhibitors of TPC2 effectively reduced EBOV infection *in vitro* (Penny et al., 2019).

Subsequently, TPC2 was found to also regulate MERS-CoV infectivity. MERS-CoV has been isolated for the first time in Saudi Arabia in 2012 and to date has affected > 1,500 individuals worldwide, with a high fatality rate (∼30–50%) (Chan et al., 2015; Memish et al., 2020). The viral S protein binds to the transmembrane receptor, dipeptidyl peptidase 4 (DPP4), and is then primed for fusion with the cell membrane by multiple proteases, including furin, trypsin, and transmembrane protease/serine subfamily member 2 (TMPRSS2) (Chan et al., 2015; V’kovski et al., 2020). In the absence of cell surface proteases, MERS-CoV entry is mediated by clathrin-mediated endocytosis followed by S protein cleavage by the endosomal cathepsins B and L (Shirato et al., 2013; Chan et al., 2015). This, in turn, triggers S protein-dependent fusion of viral and endosomal membranes, which results in the release of viral genome into the cytoplasm (Shirato et al., 2013; Chan et al., 2015). Genetic silencing of endogenous TPC1 or TPC2, but not TRPML1, reduced MERS-CoV infectivity (Gunaratne et al., 2018a). The same inhibitory effect on MERS-CoV infection was achieved by overexpressing TPC1 or TPC2 (Gunaratne et al., 2018a), a maneuver that interferes with EL trafficking. Likewise, MERS-CoV infectivity was reduced by blocking TPCs with Ca_*V*_ antagonists, i.e., nifedipine, nimodipine and nicardipine, Na_*V*_ antagonists, i.e., benzocaine and procaine, and bisbenzylisoquinoline alkaloids, including fangchinoline, thaligine, and tetrandrine (Gunaratne et al., 2018a). Fangchinoline and thaligine were the most effective drugs at impairing MERS-CoV entry into host cells and Ca^2+^ imaging confirmed that fangchinoline blocked NAADP-induced intracellular Ca^2+^ release in living cells (Gunaratne et al., 2018a). Furthermore, MERS-CoV infection was impaired by reducing PI(3,5)P_2_ levels (Gunaratne et al., 2018a), which suggests that PI(3,5)P_2_-dependent regulation of TPCs may play a crucial role in controlling MERS-CoV infection. The role of EL Ca^2+^ release in mediating this process was further suggested by the inhibitory effect on MERS-CoV infectivity of chloroquine and the weak base ammonium chloride (NH_4_Cl) (Gunaratne et al., 2018a). These drugs inhibit cathepsin activity and, therefore, prevent S protein activation, by neutralizing EL pH (Chan et al., 2015). However, an increase in EL pH also represents a widely employed strategy to deplete the EL Ca^2+^ store, as intraluminal Ca^2+^ reloading impinges on the proton-motrive force (Morgan et al., 2011; Faris et al., 2018). Furthermore, bafilomycin A1, which causes an increase in EL pH by inhibiting the v-ATPase activity and discharging the EL Ca^2+^ pool (Faris et al., 2019; Moccia et al., 2020b), may also reduce MERS-CoV infection *in vitro* (Gunaratne et al., 2018a). Therefore, TPC-mediated EL Ca^2+^ mobilization may be regarded as a crucial regulator of MERS-CoV entry in host cells.

## TPCs as Putative Targets to Prevent SARS-COV-2 Infection in the Cardiovascular System

**FIGURE 2:**
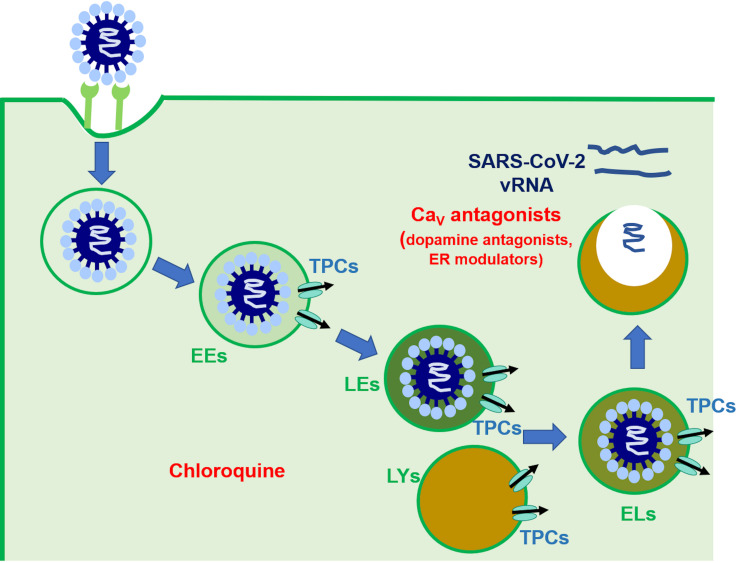
The putative role of TPCs in SARS-CoV-2 endocytosis and EL trafficking. This picture describes the mechanism whereby SARS-CoV-2 has been suggested to penetrate and replicate in host cells. It has been suggested that the endocytosis, onward trafficking, and liberation of SARS-CoV-2 is finely regulated by EL TPCs (see text for further details). The site of action of chloroquine and Ca_*V*_ antagonists [as well as dopamine antagonists and estrogen receptor (ER) modulators] were shown.

SARS-CoV-2 entry in host cells is mediated by pH-dependent endocytosis and requires proteolytic priming of the viral S protein by furin, TMPRSS2 and, to a lesser extent, cathepsins B and L (Coutard et al., 2020; Hoffmann et al., 2020; Walls et al., 2020). Following successful endocytosis, SARS-CoV-2 escapes from the EL compartment and replicates in the cytosol from which it can spread to adjacent cells (Figure 2; Coutard et al., 2020; Hoffmann et al., 2020; Walls et al., 2020). Two pieces of evidence suggest that TPCs may orchestrate SARS-CoV-2 trafficking toward LEs/lysosomes and control the release of viral mRNA into the cytosol (Figure 2). First, SARS-CoV-2 infectivity is reduced by preventing EL Ca^2+^ refilling with NH_4_Cl and chloroquine (Hoffmann et al., 2020), whereas bafilomycin A1 blocks SARS-CoV translocation (Yang and Shen, 2020). Second, SARS-CoV-2 endocytosis is inhibited by inhibiting TPCs with tetrandrine and by preventing PI(3,5)P_2_ production with PIKfyve (Ou et al., 2020). As aforementioned, tetrandrine does not selectively target TPCs and, therefore, additional studies exploiting the genetic manipulation and/or more specific blockers (e.g., NED-19) of TPCs are required to confirm their involvement in SARS-CoV-2 infection. Nevertheless, there is an emerging consensus supporting the permissive role of TPCs in SARS-CoV-2 endocytosis and EL trafficking (Chao et al., 2020; Moccia et al., 2020a; Vassileva et al., 2020; Yang and Shen, 2020). Therefore, we put forward the hypothesis that the pharmacological blockade of TPCs could represent a promising strategy to prevent/attenuate the detrimental consequences of COVID-19 on the cardiovascular system, where ACE2 is widely expressed and which can be directly infected by SARS-CoV-2. This hypothesis would further support the suggested beneficial effect of chloroquine, which may prevent EL Ca^2+^ refilling, in COVID-19 patients (Inciardi et al., 2020). Unfortunately, this drug is known to induce pro-arrhythmic events at high doses (Mazzanti et al., 2020) and more recent reports failed to validate it as an effective drug against SARS-CoV-2 (Carafoli, 2020). Nevertheless, its efficacy against viral infection endorses the pharmacological inhibition of TPC-mediated EL Ca^2+^ release as a promising therapeutic option.

## Conclusion

Herein, we discussed the hypothesis to target EL TPCs to interfere with the infection of cardiovascular cells, including cardiomyocytes, VSMCs, and vascular endothelial cells, by SARS-CoV-2. The cardiovascular system displays a dangerous sensitivity to SARS-CoV-2-dependent infection, as documented by the cardiovascular complications associated with COVID-19 and to the high incidence of infection among subjects suffering from cardiovascular comorbidities. The pharmacology of TPCs includes established Ca_*V*_ antagonists (Figure 2), that are already in use to treat cardiovascular disorders, including hypertension, acute coronary syndrome, and AMI, and has been recently expanded by novel FDA-approved drugs (Figure 2), such as dopamine antagonists and selective estrogen receptor modulators. We suggest that pre-clinical and clinical studies should investigate the protective effects of these drugs on the cardiovascular system in COVID-19 patients. The role of TPCs in SARS-CoV-2 endocytosis in cardiovascular cells remains, however, to be firmly established.

## Data Availability Statement

The original contributions presented in the study are included in the article/supplementary material, further inquiries can be directed to the corresponding author/s.

## Author Contributions

FM drafted the manuscript and supervised the work. All authors contributed to the preparation of the manuscript and approved the submitted version.

## Conflict of Interest

The authors declare that the research was conducted in the absence of any commercial or financial relationships that could be construed as a potential conflict of interest.
