# Corrigendum: Targeting Endolysosomal Two-Pore Channels to Treat Cardiovascular Disorders in the Novel COronaVIrus Disease 2019

**DOI:** 10.3389/fphys.2021.690189

**Published:** 2021-05-28

**Authors:** Francesco Moccia, Sharon Negri, Pawan Faris, Angelica Perna, Antonio De Luca, Teresa Soda, Roberto Berra-Romani, Germano Guerra

**Affiliations:** ^1^Laboratory of General Physiology, Department of Biology and Biotechnology “L. Spallanzani”, University of Pavia, Pavia, Italy; ^2^Department of Medicine and Health Sciences “V. Tiberio”, University of Molise, Campobasso, Italy; ^3^Section of Human Anatomy, Department of Mental and Physical Health and Preventive Medicine, University of Campania “Luigi Vanvitelli”, Naples, Italy; ^4^Department of Brain and Behavioral Sciences, University of Pavia, Pavia, Italy; ^5^School of Medicine, Department of Biomedicine, Benemérita Universidad Autónoma de Puebla, Puebla, Mexico

**Keywords:** SARS-CoV-2, COVID-19, cardiovascular system, two-pore channels, PI(3,5)P_2_, endolysosomal Ca^2+^ signaling, NAADP

An author name was incorrectly spelled as Roberto-Berra Romani. The correct spelling is Roberto Berra-Romani.

In the original article Penny et al. (2019) was not cited in the article. The citation has now been inserted in “4. TPCs mediate entry and trafficking of viruses in host cells” and should read:

A recent screening campaign confirmed that TPC2 activation regulates EBOV entry in host cells. Measurement of PI(3,5)P_2_-evoked lysosomal currents, NAADP-induced Ca^2+^ release and single-channel activity, revealed that FDA-approved dopamine antagonists, such as pimozide and fluphenazine, and selective estrogen receptor modulators, such as clomiphene and raloxifene, were also able to target TPC2 by plugging the channel pore (Penny et al., 2019). Furthermore, these novel inhibitors of TPC2 effectively reduced EBOV infection *in vitro* (Penny et al., 2019).

In the original article, there was an error. The assertion “Subsequently, the same group demonstrated that TPC2 also regulates MERS-CoV infectivity” is not correct as these findings were obtained by two different groups.

A correction has been made to “4. TPCs mediate entry and trafficking of viruses in host cells”:

Subsequently, TPC2 was found to also regulate MERS-CoV infectivity.

The authors apologize for this error and state that this does not change the scientific conclusions of the article in any way. The original article has been updated.
